# Effectiveness of including probiotics to *Helicobacter pylori* eradication therapies

**DOI:** 10.3164/jcbn.20-37

**Published:** 2020-06-05

**Authors:** Rieko Mukai, Osamu Handa, Yosuke Suyama, Atsushi Majima, Yuji Naito

**Affiliations:** 1 Department of Molecular Gastroenterology and Hepatology, Kyoto Prefectural University of Medicine, 465 Kajii-cho, Kamigyo-ku, Kyoto 602-8566, Japan; 2 Division of Gastroenterology, Department of Internal Medicine, Kawasaki Medical School, 577 Matsushima, Kurashiki-shi, Okayama 701-0192, Japan

**Keywords:** probiotics, eradication therapy of *Helicobacter pylori*, Miya-BM^®^

## Abstract

The eradication rate of *Helicobacter pylori* (*H. pylori*) with proton pump inhibitors, amoxicillin, and clarithromycin has reportedly decreased. Some studies have found probiotics to be useful in eradicating *H. pylori*, but these effects have not been sufficiently investigated. We aimed to elucidate the role of probiotics in eradicating *H. pylori* infection. Patients in our hospital with *H. pylori* infection that received standard treatment from January 2015 to December 2016 were retrospectively evaluated (*n* = 468). They were divided into three groups based on their treatment regime, being either proton pump inhibitors, amoxicillin, or clarithromycin (PPI group), vonoprazan, amoxicillin, or clarithromycin (VPZ group), and proton pump inhibitors, amoxicillin, or clarithromycin/probiotics (Miya-BM^®^) (PPI + MBM group). We retrospectively evaluated the *H. pylori* eradication rate and reported side effects. According to intention-to-treat analyses, the eradication rate of *H. pylori* was significantly higher in the PPI + MBM group (87.1%) than in the PPI group (70.1%). There was no difference in side effects between any of the three groups. In conclusion, Miya-BM^®^ may have an additive effect when included with eradication therapies for *H. pylori*.

## Introduction

*Helicobacter pylori* (*H. pylori*) is a known stomach carcinogen,^(1,2)^ and its eradication has been shown to reduce the incidence of stomach cancer.^(3)^ Since 2000, standard *H. pylori* eradication therapy in Japan has consisted of a combination of proton pump inhibitors (PPI), amoxicillin (AMX), and clarithromycin (CLR). However, the eradication rate for *H. pylori* with this standard primary eradication therapy has reportedly decreased,^(4)^ with this decrease thought to be correlated with the increase of anti-CLR resistance of the bacteria. Therefore, many researchers have extensively investigated antibiotics that *H. pylori* are susceptible to.

Vonoprazan (VPZ) is a novel, orally active potassium-competitive acid blocker (P-CAB) discovered and synthesized by Takeda Pharmaceutical Company Ltd., Japan. VPZ provide faster, more potent and sustained gastric acid suppression than standard PPIs. When VPZ was used instead of PPI (with the same antibiotics as mentioned above), there was an increased eradication of *H. pylori* significantly.^(5)^ However, VPZ has some concerns. We have found that VPZ may increase enteric infection risk more than PPIs by affecting intestinal microbiota. Specifically, VPZ may promote *Clostridium difficile* (*C. difficile*) infection by decreasing the genera prevent for the establishment of *C. difficile* infection such as *Blautia* and *Coprococcus*.^(6)^ In addition, VPZ has been reported to dose-dependently increase serum gastrin levels, which have been suspected of causing gastric carcinoids in an animal model.^(7)^ Although the effects of VPZ are promising, the use of PPI in *H. pylori* eradication might be recommended until the safety of VPZ is confirmed. Therefore, a PPI-based method to improve *H. pylori* eradication is essential.

In some reports, probiotics have been shown to improve the eradication rate with minimal side effects.^(8,9)^ Miya-BM^®^ (MBM) is a probiotic drug that contains *Clostridium butyricum* (*C*. butyricum) MIYAIRI 588. In a preliminary *in vitro* study, *C. butyricum* MIYAIRI 588 inhibited the growth of *H. pylori*.^(10)^ However, there are scarce reports that have examined its effectiveness in eradicating *H. pylori*. The purpose of this study is to elucidate the efficacy of MBM in the context of *H. pylori* eradication therapy.

## Materials and Methods

**Fig. 1:**
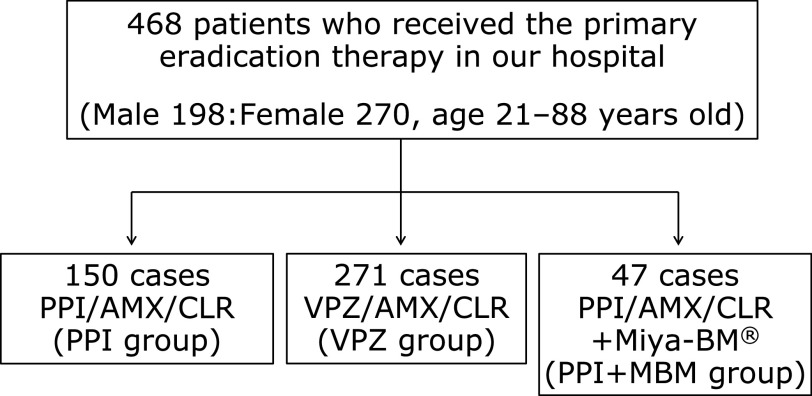
Study design. Patients who received the standard primary eradication therapy for *H. pylori* were divided into three groups based on their treatment regime, being either PPI/AMX/CLR (PPI group), VPZ/AMX/CLR (VPZ group), and PPI/AMX/CLR/Miya-BM^®^ (PPI + MBM group). PPI, proton pump inhibitors; AMX, amoxicillin; CLR, clarithromycin.

We retrospectively evaluated patients who received the standard primary eradication therapy for *H. pylori* from January 2015 to December 2016 at Kyoto Prefectural University of Medicine. Patients who dropped out were considered as treatment failures. They were divided into three groups based on their treatment regime, being either PPI/AMX/CLR (PPI group), VPZ/AMX/CLR (VPZ group), and PPI/AMX/CLR/probiotics (Miya-BM^®^; MBM) (PPI + MBM group). A cohort of 468 patients participated in this study (198 male, 270 female; age 21–88 years) and was divided into three treatment groups: PPI group (*n* = 150), VPZ group (*n* = 271), and PPI + MBM group (*n* = 47) (Fig. 1). Protocols were approved by the Medical Ethics Review Committee of the Kyoto Prefectural University of Medicine (Kyoto, Japan).

### Statistical analyses

We retrospectively evaluated the *H. pylori* eradication rate and the treatments’ side effects. Intention-to-treat (ITT) and per-protocol (PP) analyses excluded patients lost to follow-up or prematurely withdrew before completion of the study. Significant differences between the two groups were analyzed using Student’s *t* test. Differences with *p*<0.05 were considered to be statistically significant.

## Results

**Fig. 2:**
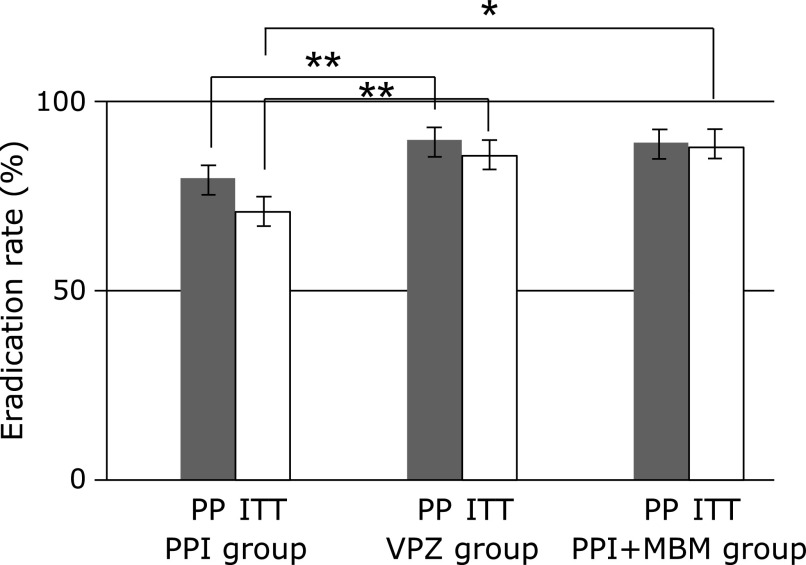
The eradication rate of three groups. In an ITT analysis, the eradication rate was 70.1% in PPI group, 84.9% in VPZ group, and 87.1% in PPI + MBM group. In a PP analysis, the eradication rate was 81.7% in PPI group, 89.8% in VPZ group, and 89.1% in PPI + MBM group. (*n* = 150 in PPI group, *n* = 271 in VPZ group, *n* = 47 in PPI + MBM group). ITT, intention-to-treat; PP, per-protocol. *p<0.05, **p<0.01.

In an ITT analysis, the eradication rates were 70.1% in the PPI group, 84.9% in the VPZ group, and 87.1% in the PPI + MBM group. In a PP analysis, the eradication rate was 81.7% in the PPI group, 89.8% in the VPZ group, and 89.1% in the PPI + MBM group (Fig. 2). There was a significant difference in the eradication rate between the PPI and VPZ groups both in ITT and PP analyses (*p* = 0.0008 in ITT analysis; *p* = 0.0073 in PP analysis). Additionally, ITT analysis of the eradication rate in the PPI + MBM group was significantly higher than that of the PPI group (*p* = 0.0195). The main side effect was diarrhea, eruption, and stomatitis. Fortunately, there were no side effects severe enough to require hospitalization. Side effects were reported in 14.7% of the PPI group, 10.0% of the VPZ group, and 19.1% of the PPI + MBM group. There was no significant difference between the three groups (Table 1).

## Discussion

In this study, we reported the additive effects of MBM, a probiotic, on *H. pylori* eradication when combined with conventional treatment regimes. So far, the most frequently studied probiotics are butyric acid bacteria, particularly *Lactobacillus sp*.^(11,12)^ During its viable bacterial preparations, butyric acid bacteria form spores that are not easily affected by antibacterial drugs even if they are not imparting artificial resistance.^(13)^ Further, it is reported that butyric acid bacteria are useful for the prevention and treatment of diarrhea.^(14)^ Therefore, we chose MBM as the probiotic for this study.

We suspect three factors that contribute to the effectiveness of MBM, the first one being its antibacterial properties. It is known that butyric acid has an antibacterial property that inhibits the growth of *H. pylori*.^(10)^ As mentioned above, butyric acid bacteria are less susceptible to antibacterial agents and can survive even if exposed to antibacterial agents by forming spores. It is also known that butyric acid bacteria continue to germinate and proliferate after the concentration of antibacterial agents in the digestive tract decreases, thereby resuming their role as a probiotic.^(14)^ Further, the butyric acid damages the integrity of the *H. pylori* cell envelope, which is necessary for many vital cell functions such as transport, peptidoglycan biosynthesis and cross-linking, as well as the synthesis of lipids. Disturbance of the cell envelope causes a destabilization of the cell membrane and, ultimately, cell death.^(15)^

The second factor that we suspect to contribute to the effectiveness of MBM is its capacity to inhibit *H. pylori* from adhering to gastric epithelial cells. Gastritis associated with *H. pylori* infection is triggered by such adhesion. The effects of *C. butyricum* on adhesion of *H. pylori* to human gastric cancer cells (MKN45) was reported by Takahashi *et al.*^(10)^ Since *C. butyricum* inhibits *H. pylori* from adhering to human gastric cancer cells, it may inhibit *H. pylori* from adhering to gastric epithelial cells in the same way.

The third factor that we suspect to contribute to the effectiveness of MBM in our study is its capacity to improve the gut microbiota. It is known that probiotic supplementation can make the gut microbiota more resilient to the effects of antibiotics. The benefits of “good” antibiotic-resistant bacteria in the gut—such as *C. butyricum*—may increase the eradication rate of *H. pylori*.^(16)^

It has been reported that probiotic supplementation, together with typical eradication therapies for *H. pylori*, reduces unpleasant side effects.^(8,17)^ However, in the present study, we found no significant differences in the prevalence of side effects between the three groups. Zheng *et al.*^(11)^ revealed that *H. pylori* eradication rates had remarkably improved with combination therapy of a lactobacillus-containing probiotic, but there was no significant decrease in adverse side effects. To our knowledge, no publications exist to date that describes in detail the mechanism by which probiotics reduce or maintain the prevalence of adverse side effects. The effect of probiotics on negative side effects is still ambiguous and requires further study.

There are some limitations to this study—first, we were limited by the relatively small sample size from one center, as well as the capacity to only investigate retrospectively. Second, the prevalence of side effects could not be consistently determined, since doctors could not have known of our intentions while they were treating their patients. Therefore, an accurate analysis may not have been performed.

Third, factors affecting the eradication rate and the ratio of drug sensitivity were not investigated in this study. For example, it is known that CYP2C19 metabolizes PPI, and the serum concentration of PPI is affected by a patient’s CYP2C19 gene polymorphism, which has been reported to affect its therapeutic effect. As a rapid metabolizer, CYP2C19 may deem our *H. pylori* eradication therapy as ineffective.^(18)^ Furthermore, an antibiotic susceptibility test by bacterial culture from each patient’s gastric biopsy is recommended to improve the eradication rate ultimately.^(19)^ It is reported that the eradication rates are dependent on the susceptibility of the strain to metronidazole and CLR,^(20)^ and the eradication rate can be improved by performing a susceptibility test and selecting an appropriate antimicrobial agent. However, in clinical practice, this procedure is not feasible and infrequently performed in Japan.

Despite these limitations, our study reported that the eradication rate of *H. pylori* was significantly increased by adding MBM with conventional PPI-based therapy when compared to that without MBM, being comparable to VPZ-based eradication therapy.

In conclusion, the supplemental use of Miya-BM^®^ in *H. pylori* eradication therapy with PPI is recommended until the safeness of VPZ will be confirmed.

## Author Contributions

YN supervised this study, and RM and OH were responsible for its conception, design, and critical revision. AM and YS contributed to data analysis and interpretation. 

## Figures and Tables

**Table 1:** Side effects of eradication therapy of *H. pylori*

	PPI group *n* = 150 (cases)	VPZ group *n* = 271 (cases)	PPI + MBM group *n* = 47 (cases)
Diarrhea	6	10	4
Eruption	7	6	2
Stomatitis	4	3	1
Heartburn, abdominal pain	2	4	1
Others	4	3	0
The incident of side effects	15.30%	9.60%	17.00%
